# Consistency of Daily Number of Reported COVID-19 Cases in 191 Countries From 2020 to 2022: Comparative Analysis of 2 Major Data Sources

**DOI:** 10.2196/65439

**Published:** 2025-02-06

**Authors:** Han Liu, Huiying Zong, Yang Yang, David C Schwebel, Bin Xie, Peishan Ning, Zhenzhen Rao, Li Li, Guoqing Hu

**Affiliations:** 1 Department of Epidemiology and Health Statistics, Hunan Provincial Key Laboratory of Clinical Epidemiology, Xiangya School of Public Health, Central South University, 172 Tongzipo Road, Yuelu District, Changsha, Hunan, 741000, China, 86 73189667218; 2 Department of Medical Records Management, Xiangya Hospital, Changsha, China; 3 Department of Statistics, Franklin College of Arts and Sciences, University of Georgia, Athens, GA, United States; 4 Department of Psychology, University of Alabama at Birmingham, Birmingham, AL, United States

**Keywords:** COVID-19, pandemic, data consistency, World Health Organization, data quality

## Abstract

**Background:**

The COVID-19 pandemic represents one of the most challenging public health emergencies in recent world history, causing about 7.07 million deaths globally by September 24, 2024. Accurate, timely, and consistent data are critical for early response to situations like the COVID-19 pandemic.

**Objective:**

This study aimed to evaluate consistency of daily reported COVID-19 cases in 191 countries from the Johns Hopkins University Center for Systems Science and Engineering (JHU CSSE) and the World Health Organization (WHO) dashboards during 2020‐2022.

**Methods:**

We retrieved data concerning new daily COVID-19 cases in 191 countries covered by both data sources from January 22, 2020, to December 31, 2022. The ratios of numbers of daily reported cases from the 2 sources were calculated to measure data consistency. We performed simple linear regression to examine significant changes in the ratio of numbers of daily reported cases during the study period.

**Results:**

Of 191 WHO member countries, only 60 displayed excellent data consistency in the number of daily reported COVID-19 cases between the WHO and JHU CSSE dashboards (mean ratio 0.9-1.1). Data consistency changed greatly across the 191 countries from 2020 to 2022 and differed across 4 types of countries, categorized by income. Data inconsistency between the 2 data sources generally decreased slightly over time, both for the 191 countries combined and within the 4 types of income-defined countries. The absolute relative difference between the 2 data sources increased in 84 countries, particularly for Malta (*R*^2^=0.25), Montenegro (*R*^2^=0.30), and the United States (*R*^2^=0.29), but it decreased significantly in 40 countries.

**Conclusions:**

The inconsistency between the 2 data sources warrants further research. Construction of public health surveillance and data collection systems for public health emergencies like the COVID-19 pandemic should be strengthened in the future.

## Introduction

The COVID-19 pandemic represents one of the greatest global public health challenges of this century. According to World Health Organization (WHO) statistics, by September 24, 2024, COVID-19 led to over 7.04 million deaths globally [1]. Critical flaws in timely data collection and reporting have hindered and misled the prevention and control of the pandemic over the past 3 years [2]. For example, during the early phase of the pandemic, the cumulative number of COVID-19 cases in the United States released by the Johns Hopkins University dashboard quickly exceeded 1 million individuals by the end of April 2020, substantially higher than governmental statistics [3]. Such discrepancies created considerable chaos for US governmental responses to the COVID-19 pandemic [4]. To better respond to future pandemics and other global health crises, it is helpful to examine and summarize important lessons we learned.

One key aspect of public health crisis and pandemic preparedness is the need for high-quality data to guide decision-making, practice, and research about prevention and control. Furthermore, 2 open-access data sources were widely used in research and decision-making about the COVID-19 pandemic [5], the COVID-19 dashboards released by the WHO and the Johns Hopkins University Center for Systems Science and Engineering (JHU CSSE). The 2 data sources collected data in different ways and have been reported as having somewhat inconsistent data in several countries [6]. However, a comprehensive evaluation of the consistency between the 2 data sources has not been conducted across multiple countries and over the key time period of the pandemic, from 2020 to 2022.

Therefore, this study systematically examined the consistency of the number for daily reported COVID-19 cases in 191 countries from 2 data sources, WHO and JHU CSSE, from 2020 to 2022.

## Methods

### Database

Given their wide use and numerous citations, we selected the publicly accessible WHO COVID-19 dashboard and the JHU CSSE COVID-19 dashboard as the major sources to investigate data patterns concerning COVID-19 pandemic infection rates [6]. The WHO dashboard was created by the WHO. In January 2020, the WHO developed the case definition of COVID-19 infection and requested all member countries to report the number of daily reported cases through the International Health Regulations national focal points. It also collected COVID-19 daily cases and death counts from internet-based public sources (public dashboards and social media) as a supplement [7]. The JHU CSSE dashboard was created by Johns Hopkins University in early January 2020. It gathered data from publicly accessible sources referenced in a version-controlled README file on the repository [5].

For this study, we retrieved publicly accessible numbers of daily reported COVID-19 cases in the 191 United Nations member countries from both data sources between January 22, 2020 and December 31, 2022. In total, there were 6 days with missing values in the 2 data sources for all 191 countries and all study days together, comprising 0.00003% (6/205,325) of all study days. Considering the small number of missing values and the fact that they were unlikely to create significant impact on our results, we excluded those days from our analysis. Data before January 22, 2020, were also not included, as the outbreak had just initiated and the JHU CSSE COVID-19 dashboard was not yet established.

### Data Analysis

In total, 2 measures were used to assess consistency between the 2 data sources. One measure was the ratio of the daily number of reported cases from the JHU CSSE dashboard to that from the WHO dashboard, aligned by calendar time. As an example, in Afghanistan on March 31, 2022, the JHU CSSE dashboard reported 264 newly reported cases and the WHO dashboard reported 220. The ratio of JHU CSSE over WHO dashboards was calculated as “264/220=1.2” for that day in Afghanistan. To facilitate our description, we refer to the ratio of daily numbers of reported cases between the 2 data sources as “daily ratio” or, simply, “ratio.”

Based on the mean ratio of the 191 countries during the full study time period, we classified the daily ratios for each country into 5 categories of consistency: excellent (0.9≤mean ratio≤1.1), high (0.8≤mean ratio<0.9 or 1.1<mean ratio≤1.2), moderate (0.7≤mean ratio<0.8 or 1.2<mean ratio≤1.3), low (0.4≤mean ratio<0.7 or 1.3<mean ratio≤1.6), and poor (mean ratio<0.4 or 1.6<mean ratio). Countries where the 2 dashboards had higher consistency yielded ratios closer to 1.0, of course, and countries where there were greater discrepancies between the 2 dashboards had ratios either far below or far above 1.0.

To address certain unusual cases, we empirically built the following rules to calculate the daily ratio in certain unusual cases: (1) when the numerator and denominator were both 0, we defined the ratio as 1, as this represented perfect agreement between the dashboards; (2) when only the numerator was 0 and the denominator was 10 or greater, we defined the ratio as 0.5, and when the denominator was less than 10, we used the fourth criterion listed below to define the value of the ratio; (3) when the denominator was 0 and numerator was 10 or greater, we defined the ratio as 2.0, and when the numerator was less than 10, we used the fourth criterion below to define the value of the ratio; and (4) when the numerator and denominator were both less than 10 and their differences were less than 3 (explaining 0.1% of all data), between 4 and 6 (explaining 0.3%), or ≥7 (explaining 0.5%), we defined the ratio as 0.9 or 1.1, 0.7 or 1.3, 0.5 or 1.5, respectively, thus avoiding extremely large or small ratios due to small numerators or denominators. We mapped the mean and coefficient of variation (CV) of the daily ratios between the 2 data sources for each of the 191 countries.

We graphed box plots and performed the Kruskal-Wallis rank sum test to display and compare differences in the mean and CV of the daily ratios across groups of countries defined by income levels. The 191 countries were classified into 4 income-based categories according to the World Bank Analytical Classifications of 2023 [8]: low-income countries, lower middle-income countries, upper middle-income countries, and higher-income countries. In addition, simple linear regression, with the absolute relative difference in daily number used as the dependent variable and time (date) as the independent variable, was performed to examine trends in the daily differences during the study period. A significant and positive regression coefficient indicates worsening data consistency over time, while a significant and negative regression coefficient indicates improved data consistency.

SPSS statistics 26 (IBM Corp), R version 4.3.0 (R Core Team), and Microsoft Excel spreadsheet 2021 were used for data analysis. *P*<.05 was regarded as statistically significant.

### Ethical Considerations

This study does not cause harm to humans and does not involve individual information or commercial interest information. The data used in this study cannot be used to identify individual information, either directly or indirectly. Based on the given declarations, this study complies with the fundamental principles expressed in the Declaration of Helsinki and meets the ethical exemption requirements of the Ethical Review Measures for Life Sciences and Medical Research Involving Humans promulgated by China. Therefore, this research was exempted from ethical review.

## Results

**Figure 1.**
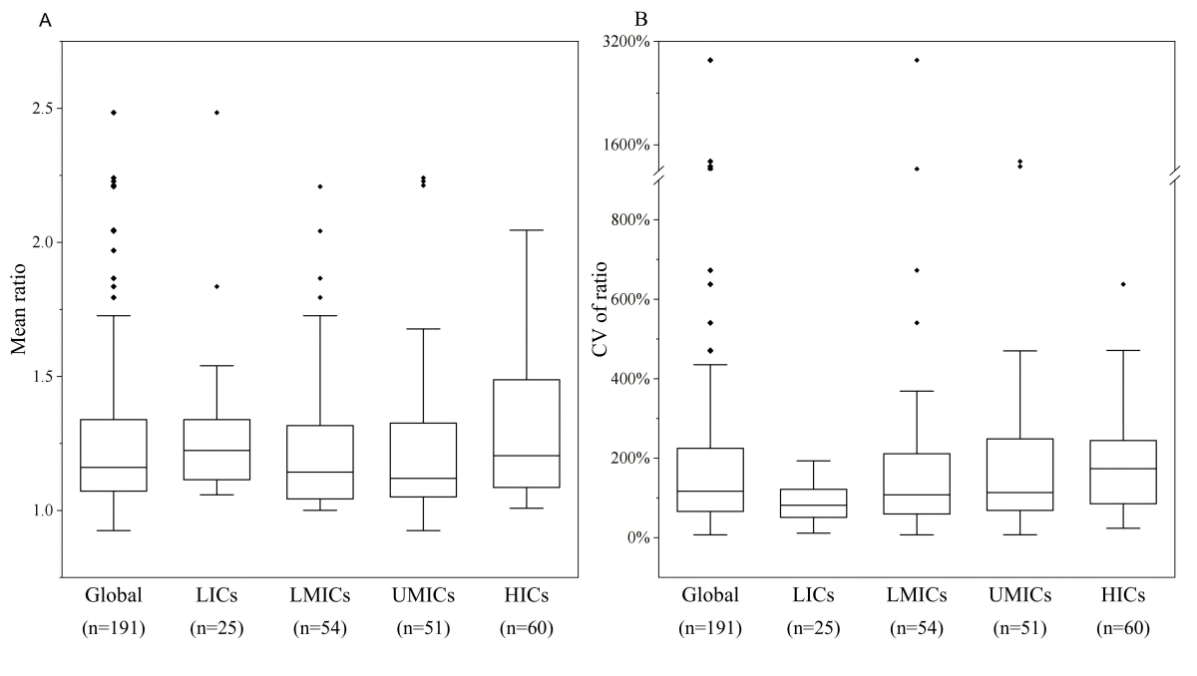
Ratio of number of daily new cases from the Johns Hopkins University Center for Systems Science and Engineering COVID-19 dashboard and from the World Health Organization COVID-19 dashboard by country income category, from January 22, 2020, to December 31, 2022. CV: coefficient of variation; HIC: higher-income country; LIC: low-income country; LMIC: lower middle-income country; UMIC: upper middle-income country.

For all observed dates combined, the median ratio between the daily number of reported COVID-19 cases in the 2 data sources for the 191 countries was 1.14 (IQR 1.07-1.34), with the lowest mean ratio in Turkey (0.92) and the highest mean ratio in Sudan (2.48) (Figure 1A). The ratios did not differ significantly across the 4 categories of countries organized by income (*P*=.12). The median CV of ratio between the daily number of reported COVID-19 cases from the 2 data sources was 1.16 (IQR 0.66-2.25) for the 191 countries. The 4 types of countries had significantly different CVs of ratio (*H*=10.08, *P*=.02) (Figure 1B).

**Figure 2.**
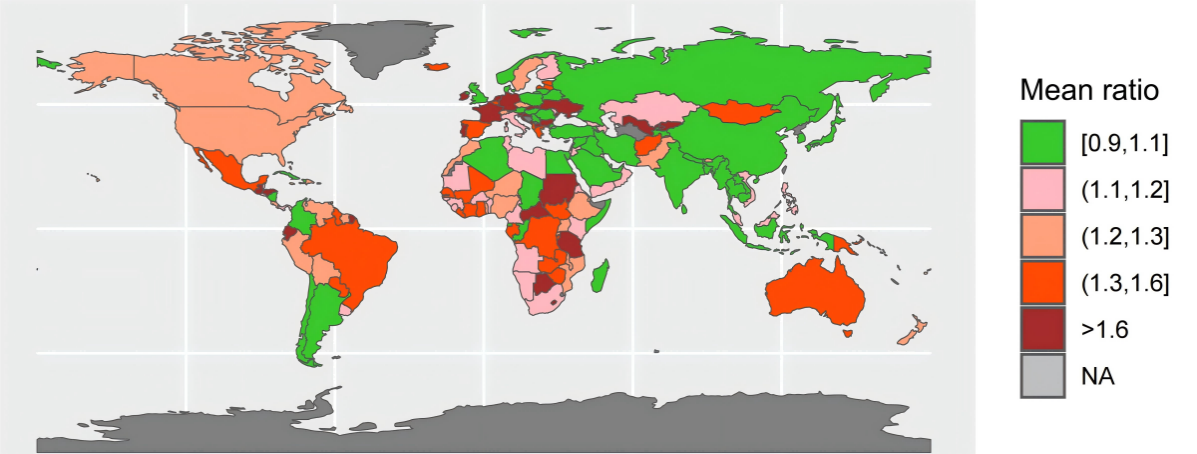
Mean ratio of number of daily new cases from the Johns Hopkins University Center for Systems Science and Engineering COVID-19 dashboard and from the World Health Organization COVID-19 dashboard across 191 countries from January 22, 2020, to December 31, 2022. The ratio was calculated as the number of daily new cases from the Johns Hopkins University Center for Systems Science and Engineering COVID-19 dashboard divided by the number from the World Health Organization COVID-19 dashboard, for each country. NA: not available.

**Figure 3.**
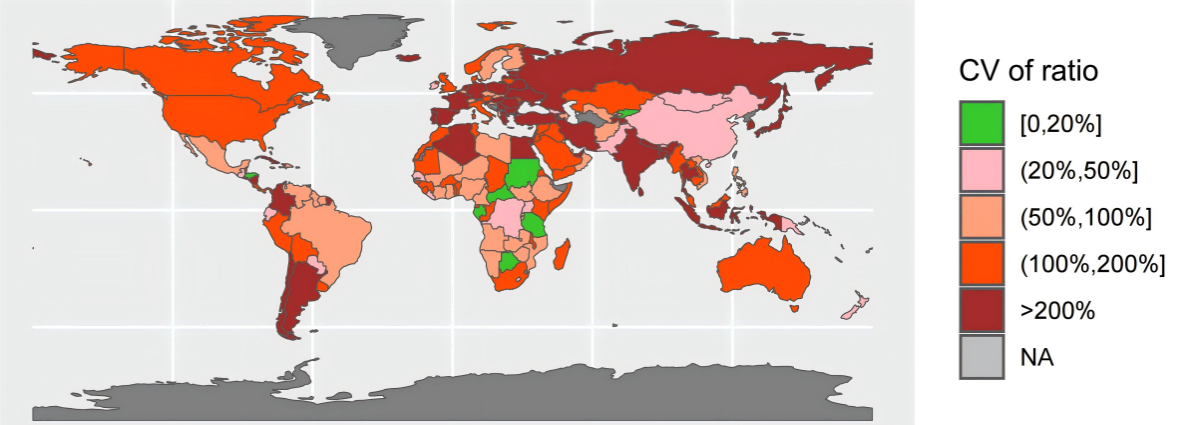
CV of ratio of number of daily new cases from the Johns Hopkins University Center for Systems Science and Engineering COVID-19 dashboard and from the World Health Organization COVID-19 dashboard across 191 countries from January 22, 2020, to December 31, 2022. The ratio was calculated as the number of daily new cases from the Johns Hopkins University Center for Systems Science and Engineering COVID-19 dashboard divided by the number from the World Health Organization COVID-19 dashboard, for each country. CV: coefficient of variation; NA: not available.

In Figure 1, the ratio was calculated as the number of daily new cases from the JHU CSSE COVID-19 dashboard divided by the number from the WHO COVID-19 dashboard, for each country. Figure 2 and Figure 3 display the variations in mean and CV of ratio between the daily number of reported COVID-19 cases from the 2 data sources across the countries. Of the 191 countries, 60 had excellent consistency (mean ratio: 0.9‐1.1), while 37 had low consistency (mean ratio: 0.4‐0.7 or 1.3‐1.6) and 23 had poor consistency (mean ratio:<0.4 or >1.6) (Figure 2). Most African and European countries had low or poor data consistency and most Asian countries had high or excellent data consistency. Strikingly, the CV of ratio between the number of daily reported COVID-19 cases from the 2 data sources during the study time period was higher than 100% in 110 countries (110/191, 57%), and 20% or less in only 8 countries (8/191, 4%) (Figure 3).

For the 191 countries combined and within all 4 income-defined types of countries, the absolute relative difference between daily number of reported COVID-19 cases from the 2 data sources all increased slightly over the study time period, with *R*^2^ values of 0.16, 0.08, 0.06, 0.14, and 0.14, respectively (Table 1). Country-specific analyses showed that the absolute relative difference increased in 84 countries but decreased in 40 countries significantly (Table 2). Notably, data inconsistency between the 2 data sources increased significantly over time in 3 countries (Malta: *R*^2^=0.25; Montenegro: *R*^2^=0.30; United States: *R*^2^=0.29).

**Table 1.** Linear trends in ratio of number of daily reported cases from the Johns Hopkins University Center for Systems Science and Engineering COVID-19 dashboard and from the World Health Organization COVID-19 dashboard from January 22, 2020, to December 31, 2022, by country income category (dependent and independent variables of linear regression model were “the absolute value of (ratio-1)” and “time (date),” respectively).

Country income	Linear regression model	Coefficient of determination (*R*^2^)	*P* value
Global	$\hat{y}$=−713.80+0.00029*x*	0.16	<.001
LICs^a^	$\hat{y}$=−1110.18+0.00045*x*	0.08	<.001
LMICs^b^	$\hat{y}$=−1025.15+0.00041*x*	0.06	<.001
UMICs^c^	$\hat{y}$=−781.22+0.00031*x*	0.14	<.001
HICs^d^	$\hat{y}$=−1453.78+0.00059*x*	0.14	<.001

a LIC: low-income country.

b LMIC: lower middle-income country.

c UMIC: upper middle-income country.

d HIC: higher-income country.

**Table 2.** Linear trends in ratio of country-specific number of daily new cases from the Johns Hopkins University Center for Systems Science and Engineering COVID-19 dashboard and from the World Health Organization COVID-19 dashboard from January 22, 2020, to December 31, 2022. Based on coefficient of determination (*R*^2^) of linear regression models with statistical significance, we classified linear trends of ratio into 3 grades: substantially (*R*^2^≥0.25), moderately (0.10<*R*^2^<0.25), and slightly (*R*^2^≤0.10). In addition, we did not detect significant changes in data consistency for 67 countries).

Linear trend		Country
**Increased (n=84)**		
	Substantially (n=3)	Malta (*R*^2^=0.25), Montenegro (*R*^2^=0.30), United States (*R*^2^=0.29)
	Moderately (n=4)	Australia (*R*^2^=0.18), Bulgaria (*R*^2^=0.11), Iran (*R*^2^=0.13), Slovenia (*R*^2^=0.21)
	Slightly (n=77)	Albania, Algeria, Antigua and Barbuda, Azerbaijan, Bahamas, Bangladesh, Barbados, Belgium, Bhutan, Bolivia, Bosnia and Herzegovina, Brazil, Brunei, Burundi, Cambodia, Canada, Chile, Comoros, Croatia, Cyprus, Czechia, Democratic Republic of Congo, Denmark, Dominica, Dominican Republic, Ecuador, Ethiopia, Fiji, Finland, Georgia, Grenada, Haiti, India, Iraq, Ireland, Italy, Kazakhstan, Kenya, Kiribati, Kuwait, Laos, Latvia, Liechtenstein, Lithuania, Maldives, Marshall Islands, Mauritius, Mexico, Micronesia, Monaco, Mongolia, Morocco, Namibia, Nauru, Norway, Palau, Panama, Papua New Guinea, Peru, Romania, Saint Kitts and Nevis, Samoa, San Marino, Saudi Arabia, Seychelles, Slovakia, Solomon Islands, South Africa, Sweden, Switzerland, Thailand, Timor, Tonga, Ukraine, United Kingdom, Vanuatu, Venezuela
**Decreased (n=40)**		
	Moderately (n=2)	Poland (*R*^2^=0.17), Spain (*R*^2^=0.11)
	Slightly (n=38)	Andorra, Argentina, Bahrain, Belarus, Burkina Faso, Cameroon, Chad, Congo, Djibouti, Egypt, El Salvador, Estonia, Germany, Greece, Guinea, Hungary, Japan, Jordan, Madagascar, Malaysia, Mali, Mauritania, Moldova, Myanmar, Netherlands, North Macedonia, Portugal, Russia, Sierra Leone, Somalia, Togo, Tunisia, Turkey, Tuvalu, United Arab Emirates, Uruguay, Yemen, Zambia
No significant change (n=67)		Afghanistan, Angola, Armenia, Austria, Belize, Benin, Botswana, Cape Verde, Central African Republic, China, Colombia, Costa Rica, Cote d’Ivoire, Cuba, Equatorial Guinea, Eritrea, Eswatini, France, Gabon, Gambia, Ghana, Guatemala, Guinea-Bissau, Guyana, Honduras, Iceland, Indonesia, Israel, Jamaica, Kyrgyzstan, Lebanon, Lesotho, Liberia, Libya, Luxembourg, Malawi, Mozambique, Nepal, New Zealand, Nicaragua, Niger, Nigeria, Oman, Pakistan, Paraguay, Philippines, Qatar, Republic of Korea, Rwanda, Saint Lucia, Saint Vincent and the Grenadines, Sao Tome and Principe, Senegal, Serbia, Singapore, South Sudan, Sudan, Suriname, Sri Lanka, Syria, Tajikistan, Tanzania, Trinidad and Tobago, Uganda, Uzbekistan, Vietnam, Zimbabwe

## Discussion

### Main Findings

This study systematically examined the consistency of number of daily reported COVID-19 cases and number-based epidemic cycles in 191 countries from the 2 most cited data sources between 2020 and 2022. We generated 2 key findings. First, there was a difference in global number of daily reported COVID-19 cases between the WHO and JHU CSSE data, and data consistency differed between the 4 categories of income-defined countries and across the 191 countries. Second, data inconsistency between the 2 data sources generally decreased slightly over time, both for the 191 countries combined and within the 4 types of income-defined countries, but it changed differently over time across the 191 individual countries.

### Interpretation of Findings

Inconsistencies in the number of daily reported COVID-19 cases between the WHO and JHU CSSE data likely reflect differences in data collection and data release strategies across the 2 data sources. The WHO data came primarily from official daily counts reported by WHO member states, territories, and areas [7], while the JHU CSSE data were derived from a combination of more than 400 sources for over 3500-point locations [5]. Compared with the WHO data, the JHU CSSE data were collected from a higher number of sources, potentially explaining why the number of daily reported COVID-19 cases from the JHU CSSE data was greater than that from the WHO data for the whole world, the 4 types of income-defined countries, and most individual countries during most dates [9]. The 2 data sources also adopted different data release strategies. The JHU CSSE COVID-19 dashboard used automated methods to extract data from each source every half hour and therefore updated data hourly [5]. In contrast, the WHO COVID-19 dashboard relied on official reports from all WHO member countries, territories, and areas, which were more prone to be delayed and less frequently updated than the JHU dashboard data [10].

Variations in data inconsistency between the WHO and JHU CSSE data might also be due to diverse data publishing policies and the evolution of data-releasing agencies (eg, delayed reporting, data entry errors, and data collection strategies), particularly at the local level across the 191 countries. As the pandemic eased, some countries like Sweden reduced the frequency of data release and even stopped publishing epidemic data, particularly at the local level [11]. These practices likely affected the original data collected by the WHO and the JHU CSSE COVID-19 dashboards in different ways. In particular, sources indexed by the JHU CSSE dashboard were more extensive and were more likely to change over time than those covered by the WHO dashboard [12].

### Implications and Limitations

Our findings have 2 important implications. First, we do not have clear empirical basis to explain the drivers behind inconsistencies in the number of COVID-19 cases between the WHO and JHU CSSE dashboards or the changes in data inconsistency over time. A variety of internal and external factors likely could have contributed to the magnitude and pattern of the inconsistency, including but not limited to changes in government intervention policies or testing capacities.

Policy makers, researchers, and public health practitioners should recognize that the 2 data sources generate somewhat different and even occasionally conflicting results when they use the historical COVID-19 data collected by the WHO and JHU CSSE dashboards to summarize pandemic control experiences and lessons, conduct historical trend analyses, and evaluate intervention strategies [13-15]. Because strong evidence and detailed guidance are lacking concerning which of the 2 data sources is more valid for which specific time periods and countries, we recommend users consider both, recognizing each may have flaws. It also would be valuable to conduct further research assessing the quality of the data sources. For example, comparisons of the data sources with known cycles of the COVID-19 epidemic may yield additional information about the validity of each data source [16,17]. Another useful approach to bridge the gaps in high-level data from mainstream sources is to cross-validate them with data provided by local health authorities whenever and wherever available. This may be particularly useful for data on hospitalization and on fatalities, which are less sensitive to testing policies, health care–seeking behavior, and reporting bias [18].

Second, our results highlight the importance of gathering consistent and accurate data to fight against future pandemics or other public health emergencies [19]. Inconsistent data can lead to undesired consequences in decision-making, research, and prevention efforts [20,21]. Development of standardized data collection and release protocols, along with infrastructure- and competency-building to help all countries gather accurate health data, will prepare us not just for current health situations but also for future pandemics and broad public health emergencies [22].

Our research is primarily limited by the unavailability of a gold standard criterion. We assessed the consistency of 2 data sources and cannot comprehensively assess the reliability or validity of either, or identify which might be more accurate. No alternative or recognized valid measure of COVID-19 infections or fatalities exists worldwide. In addition, due to the unavailability of detailed information about COVID-19 data collection methods or data release policies across the 191 countries, we cannot reasonably interpret exactly why data consistency variations emerged across the 191 countries or why there were significant increases or decreases in data consistency over time for some countries. Future research may consider innovative strategies to identify key factors contributing to the observed data inconsistencies and significant changes in data consistency. Hospitalization data might be considered as a surrogate gold standard, for example, to approximate accuracy of other data sources [23], perhaps providing valuable clues. Advanced data analysis strategies, such as data harmonization or deep machine learning, might also help uncover reasons behind the results we report and be useful to address data accuracy in future public health emergencies. Finally, evidence-based data collection and release guidelines should be established by the WHO or other governing bodies, perhaps supplemented with financial and technical support to those countries with limited resources to follow international guidelines. With strong international collaboration, we should be able to improve transparency, consistency and overall quality of data emerging from public health crises in the future.

### Conclusions

We report a moderate difference in global average daily number of reported COVID-19 cases between 2020 and 2022 in the WHO and JHU CSSE datasets. The COVID-19 data inconsistency changed significantly across the countries studied and across time, both globally and in 124 individual countries. Data users should be cautious in interpreting the data, given inconsistency across the 2 most commonly cited COVID-19 data sources. Further research is recommended to develop guidelines to support valid interpretation of existing COVID-19 data, explore possible reasons for discrepancies, and understand factors such as governmental policies and data collection systems that may lead to data inconsistency. With those efforts, society can be best prepared to conduct valid health-related data collection in response to future pandemics and public health emergencies.

## Abbreviations

CV: coefficient of variation
JHU CSSE: Johns Hopkins University Center for Systems Science and Engineering
WHO: World Health Organization
